# PharmFrag: An Easy and Fast Multiplex Pharmacogenetics Assay to Simultaneously Analyze 9 Genetic Polymorphisms Involved in Response Variability of Anticancer Drugs

**DOI:** 10.3390/ijms21249650

**Published:** 2020-12-17

**Authors:** Régis Bouvet, Marie-Clémence Verdier, Yahya El Baroudi, Marie-Dominique Galibert, Véronique David, Sacha Schutz, Camille Tron

**Affiliations:** 1 Department of Molecular Genetics and Genomics, Rennes University Hospital, 35000 Rennes, France; regis.bouvet@chu-rennes.fr (R.B.); Yahya.ElBaroudi@chu-rennes.fr (Y.E.B.); Marie-Dominique.GALIBERT.ANNE@chu-rennes.fr (M.-D.G.); veronique.david@chu-rennes.fr (V.D.); sacha@labsquare.org (S.S.); 2 IRSET (Institut de Recherche en Santé, Environnement et Travail), University of Rennes, CHU Rennes, EHESP, UMR_S 1085, 35000 Rennes, France; Marie-clemence.verdier@chu-rennes.fr; 3 Inserm, Centre D’investigation Clinique 1414, Rennes University Hospital, 35000 Rennes, France; 4 Pharmacology Department, Rennes University Hospital, 35000 Rennes, France; 5 Genetic Laboratory Department, Brest University Hospital, 29200 Brest, France

**Keywords:** pharmacogenetics, multiplex, anticancer drug

## Abstract

Regarding several cytotoxic agents, it was evidenced that genetic polymorphisms in genes encoding enzymes involved in their metabolism are associated with higher risk of toxicity. Genotyping these genes before treatment is a valuable strategy to prevent side effects and to predict individual response to drug therapy. This pharmacogenetic approach is recommended for chemotherapies such as thiopurines (azathioprine, 6-mercaptopurine, thioguanine), irinotecan, and fluoropyrimidines (capecitabine and 5-fluorouracil). In this study, we aimed at developing and validating a fast, cost-effective, and easily implementable multiplex genotyping method suitable for analyzing a panel of nine variants involved in the pharmacogenetics of widely prescribed anticancer drugs. We designed a multiplex-specific PCR assay where fragments were labeled by two different fluorescent dye markers (HEX/FAM) identifiable by fragment analysis. These two labels were used to discriminate bi-allelic variants, while the size of the fragment allowed the identification of a particular polymorphism location. Variants of interest were *TPMT* (rs1800462, rs1142345, rs1800460), *NUDT15* (rs116855232), *DPYD* (rs55886062, rs3918290, rs67376798, rs75017182), and *UGT1A1* (rs8175347). The assay was repeatable, and genotypes could be determined when DNA sample amounts ranged from 25 to 100 ng. Primers and dye remained stable in a ready-to-use mixture solution after five freeze–thaw cycles. Accuracy was evidenced by the consistency of 187 genotyping results obtained with our multiplex assay and a reference method. The developed method is fast and cost-effective in simultaneously identifying nine variants involved in the pharmacological response of anticancer drugs. This assay can be easily implemented in laboratories for widespread access to pharmacogenetics in clinical practice.

## 1. Introduction

In the era of personalized medicine, cytotoxic anticancer drugs remain widely used to treat hematologic malignancies and solid tumors. An important interindividual variability in drug response can be observed with these therapeutic agents. Many chemotherapies have a narrow therapeutic range; therefore, a part of this suboptimal response can be explained by variations of drug blood concentrations. Regarding several cytotoxic agents, it was evidenced that genetic polymorphisms in genes encoding enzymes involved in their metabolism are associated with blood overexposure, leading to higher risk of toxicity [1]. Genotyping these genes before treatment is a valuable strategy to prevent side effects and to predict individual response to drug therapy. Indeed, it allows the identification of patients who are carriers of allelic variants and who need dosage adjustment. This pharmacogenetic approach is strongly recommended by international clinical and pharmacological consortiums for chemotherapies, such as thiopurines (azathioprine, 6-mercaptopurine, thioguanine), irinotecan, and fluoropyrimidines (capecitabine and 5-fluorouracil) [2,3,4]. Indeed, dosing algorithms are now available to help clinicians to individualize the prescription of these drugs, taking into account genotypes of *TPMT*, *NUDT15, DPYD*, and *UGT1A1*, whose corresponding proteins metabolize thiopurines, fluoropyrimidines, and irinotecan, respectively. Thus, the implementation of pharmacogenetic assays may prevent the severe hematologic or digestive toxicities of these anticancer drugs. Routinely, laboratories only need to look for the most common and clinically relevant variants associated with the enzyme dysfunction. For *TPMT,* there are three main single-nucleotide polymorphisms (SNPs) of interest and one for NUDT15 [2]. For *DPYD*, it is recommended that four SNPs be studied, and for *UGT1A1*, a repetition in the TATA box of the promoter (allele *28) is the variant usually studied [1]. The genotype–phenotype relationships of these genetic polymorphisms are presented in Table 1. Many genotyping methods have been reported to perform these pharmacogenetic analyses [5]. First, low-throughput technologies or simplex methods are available to separately analyze the above-mentioned genetic polymorphisms (i.e., Sanger sequencing, PCR-RFLP, TaqMan^TM^ genotyping assays). However, it can be challenging and cumbersome for some laboratories to use these methods to perform multiple gene analysis. To remove this hurdle, an alternative can be to use high-throughput technologies such as microarrays and next-generation sequencing (NGS). These methods allow multiplexing to analyze panels of many genes and many samples in a single experiment. Nevertheless, result interpretations require specialized skills (e.g., bioinformatics) and are not cost-effective below a certain threshold of samples to be analyzed.

In this context, we aimed at developing and validating a fast, cost-effective, and easily implementable multiplex genotyping method suitable for analyzing a panel of nine variants involved in the pharmacogenetics of widely prescribed anticancer drugs.

## 2. Results

### 2.1. Multiplex PCR Protocol Optimization

The most important parameter of the multiplex PCR protocol is the primer concentration, which influences the intensity of the fluorescence and consequently the height of the peaks on fragment analysis. Initially, amplification reactions were performed using equal concentrations of the primers. Subsequently, the concentration of each primer was adjusted to give a more comparable peak height. Then, the primer concentrations were optimized to get signals at least 10 times higher than the background noise (raw intensity > 100). The number of PCR cycles and the DNA concentration were also adjusted. Eventually, the final procedure was chosen to obtain a ratio between intensities of the dyes FAM/HEX ≈ 1 for all polymorphism positions of interest.

**Figure 1:**
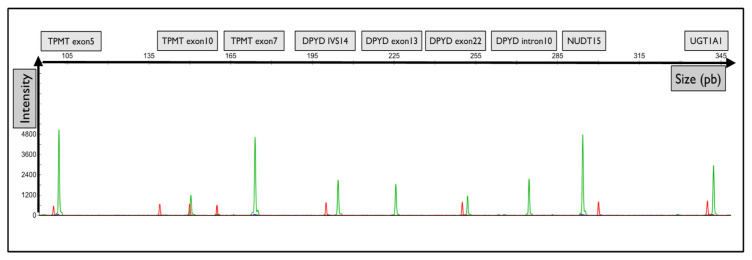
Representative graphical results of the migration of fragments from each gene of interest. Graphical electropherogram obtained for wild-type samples for all alleles tested. The green peak corresponds to the fluorescence of the HEX probe. The red peak corresponds to the size of the internal standard.

Moreover, the primers were designed with a substitution of the wild-type matrix at the -4 nucleotide of the 3′ flanking forward primers to reduce false positive genotyping and increase the assay specificity. A representative electropherogram of the migration of the fragments of interest is shown in Figure 1.

**Figure 2:**
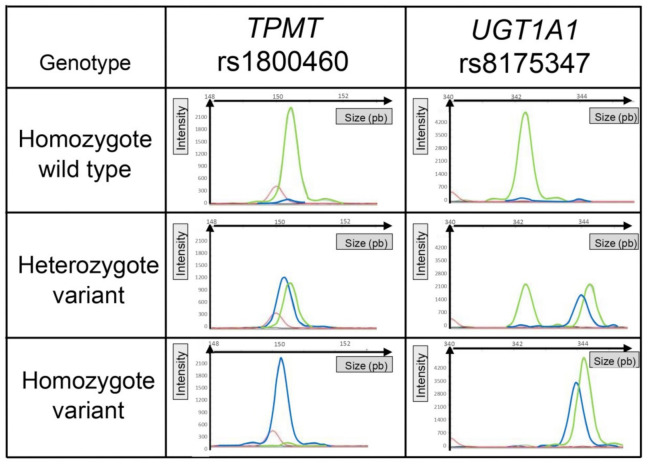
Representative graphical results of the migration of fragments according to genotype. *TPMT* rs1800460 illustrated results of allelic discrimination for a single-nucleotide polymorphism. *UGT1A1* illustrated results of allelic discrimination for a polymorphism based on a nucleotide repetition. The red peak corresponds to the size of the internal standard. The green peak corresponds to the fluorescence of the HEX probe (wild type), and the blue peak corresponds to the fluorescence of the FAM probe (variant).

Besides, all the genetic polymorphisms analyzed are DNA substitutions (SNPs) except for *UGT1A1*, which is a repetition of 6 TA nucleotides for the wild type or 7 TA for the variant. This implies that the variant forward primer *UGT1A1* amplifies only the variant DNA and that the wild-type forward primer *UGT1A1* amplifies the wild-type and variant DNA. Consequently, for the heterozygous genotype (TA6/TA7), high-resolution capillary electrophoresis was needed to distinguish the migration of two fragments associated with the same fluorescent dye and whose lengths were very close (only two base pairs different). Representative electropherograms of each genotype, for an SNP (e.g., *TPMT* rs1800460) or a repetition (e.g., *UGT1A1* rs8175347), are illustrated in Figure 2.

### 2.2. Validation of the Protocol

#### 2.2.1. Repeatability

Repeated analysis of the same internal control DNA samples (50 ng) gave the same genotype results on intraday experiments (duplicate) and interday experiments (6 days).

#### 2.2.2. Accuracy

A cohort of 187 DNA samples was screened by the multiplex protocol to check whether the genotyping results matched with the expected genotypes. All results were confirmed without false positive or false negative. The consistency of the genotyping results of these samples is reported in Table 2.

#### 2.2.3. Robustness and Intersample Contamination

The assay was designed to be performed on 50 ng of extracted DNA. However, the influence of lower and higher DNA amount on the performance of the analysis was assessed. The results are reported in Table 3. It appears that the genotypes can be accurately determined when DNA sample amounts range from 25 to 100 ng. Outside this range, the intensity of the signal does not meet the acceptance criteria to interpret migration fragment data (peak height < 100 units of intensity or saturation of the signal). No influence of the quality of the DNA was observed. Indeed, genotyping was successfully performed in our cohort, whose absorbance ratio ranged from 0.35 to 2.3 and 1.5 to 2.1 for ratios of 260/230 nm and 260/280 nm, respectively. Moreover, no intersample contamination was observed since no signal was detected at the position of the blank samples inserted between the DNA samples (data not shown).

#### 2.2.4. Stability

The stability of the ready-to-use pool of primer and fluorescent probes at the working conditions was validated for five freeze–thaw cycles. Indeed, the genotyping results were similar in an experiment performed with a freshly prepared pool and in another experiment performed with an aliquot frozen and thawed five times. Although the peak intensities of fragments were lower when a freeze-thawed reagent was used, they remained >100 intensity units (Table 4). Thus, a pool of primers can be stored at −20 °C and used several times, which is very cost saving and convenient.

## 3. Material and Methods

### 3.1. Samples and DNA Extraction

The samples analyzed in this study were anonymized DNA leftover samples from our center DNA bank. DNA was extracted from blood collected either on EDTA or heparinized tubes in humans who had given written consent for genetic research beforehand. The study was approved by a local ethical committee (authorization no. 20.131), approved on the 23 October 2020. DNA was extracted from blood with a Microlab STAR Liquid Handling System (Hamilton, Courtaboeuf, France) using a Macherey-Nagel^®^ (Hoerdt, France) Nucleospin Blood L kit as described in the manufacturer’s protocol. DNA concentration was measured using a NanoDrop One spectrophotometer (NanoDrop Technologies Inc., Wilmington, DE, USA).

### 3.2. Reference Method

All the samples used to validate the assay were previously genotyped for SNPs of interest using a routine method based on Taqman^TM^ allelic discrimination. Briefly, each SNP was analyzed using the appropriate reaction mix prepared with TaqMan^TM^ Drug Metabolism Genotyping Assays (Thermo Fisher, Waltham, MA, USA). Analysis was performed on an ABI 7900HT instrument (Applied Biosystems, Foster City, CA, USA). For the UGT1A1 *28 allele, pyrosequencing was the reference method.

### 3.3. Principle of the Assay and Design of the Primers

**Figure 3:**
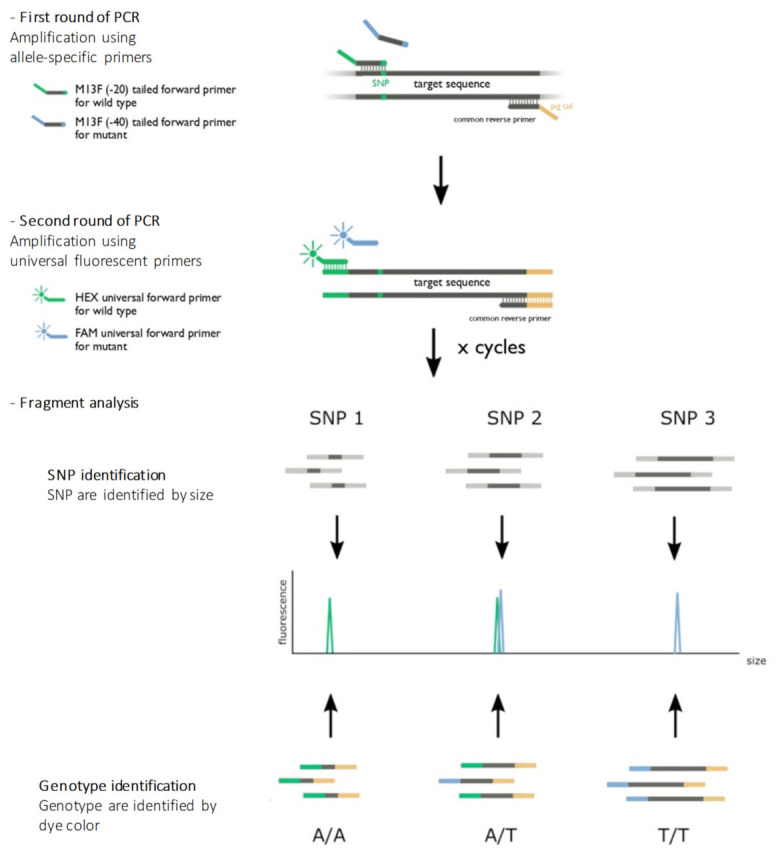
Principle of the assay. The first round of amplification used allele-specific primers designed to obtain different fragment sizes for each genetic location. The last nucleotide of the 3′ flanking forward primers was specific to the genotype (wild-type or variant allele). The reverse primer was the same for both alleles (wild type or variant allele). A pigtail GTTTCTT was added to the 5′ flanking reverse primers to improve the amplicon migration. A second round of amplification used universal primers combined with fluorescent probes. An M13 sequence (-20) GTAAAACGACGGCCAGT in the 5′ flanking forward wild-type primers allowed the hybridization and amplification of the wild-type allele, and an M13 sequence (-40) GTTTTCCCAGTCACGAC in the 5′ flanking forward variant primers allowed the hybridization and amplification of the variant allele. After denaturation, PCR products were loaded on a sequencer. The color of the fluorescence of the fragment allowed allelic discrimination, and the size of the fragment allowed genetic position discrimination.

The assay was adapted from Schuelke’s work [7]. We have designed and developed a multiplex allele-specific PCR where fragments are labeled by two different fluorescent markers (HEX/FAM) identifiable by fragment analysis. These two labels are used to characterize the genotype by discriminating bi-allelic variants, while the size of the fragment allows the identification of a particular SNP. The principle of the assay is illustrated in Figure 3.

The nine variant genomic regions were used as a reference for the selection of the primers using a Primer-BLAST tool. The primers were designed to obtain different fragment sizes for each genetic location (primer sizes varied in at least 20 nucleotides). The last nucleotide of the 3′ flanking forward primers was specific to the genotype (wild-type or variant allele). A second substitution of the wild-type matrix was applied to the -4 nucleotide of the 3′ flanking forward primers to enhance its specificity. A universal M13 sequence (−20) GTAAAACGACGGCCAGT was added to the 5′ flanking forward wild-type primers, and an M13 sequence (−40) GTTTTCCCAGTCACGAC was added to the 5′ flanking forward variant primers. A pigtail GTTTCTT was added to the 5′ flanking reverse primers to improve the amplicon migration on the capillary system and to avoid double peaks. Primer sequences are reported in Table 5.

### 3.4. PCR Multiplex Amplification

The nine variants were amplified simultaneously using a Qiagen^®^ (Courtaboeuf, France) Multiplex PCR kit following the manufacturer’s protocol. Briefly, 50 ng of DNA was mixed with a pool solution including every primer (Table 5). Fluorescent probes (HEX specific to M13 (−20) and FAM specific to M13 (−40), both at 0.7 pmol/µL in the final mix) were also added to the pool of primers (probes were provided by Eurofins MWG Operon, Les Ulis, France). Each primer concentration was optimized to get optimal signal detection (Table 5). The PCR was run on a thermocycler (Veriti™ 96-Well Thermal Cycler, Thermo Fisher Scientific, Illkirch, France) and started with an activation cycle of 95 °C for 15 min, and then 30 cycles of amplifications were run with the following sequence: 94 °C for 30 s, 58 °C for 90 s, and 72 °C for 60 s. The last cycle was set at 72 °C for 30 min.

### 3.5. Genotyping and Fragment Analysis

The PCR products were denatured with deionized formamide (Thermo Fisher Scientific, Illkirch, France) and separated on an ABI 3130 Genetic Analyzer (Applied Biosystems^TM^/Thermo Fisher Scientific, Illkirch, France). GeneScan™ 500 ROX™ (Applied Biosystems^TM^/Thermo Fisher Scientific, Illkirch, France) was used as a dye size internal standard. Data were processed using the GeneMapper^®^ 4.0 software (Applied Biosystems^TM^). We defined the acceptance criterion of the genotyping results after migration on the sequencer as follows: the height of the signal of each fragment should be higher than 100 (intensity unit) and lower than 7500 (intensity unit) to avoid saturation, and the length of each fragment should not differ from a +/− 1 base pair from the theoretical length of the fragment.

### 3.6. Internal Control Samples

In order to check the success of each set of experiments during method development and validation, DNA samples of known genotypes were used as homozygous wild-type, heterozygous, and homozygous variant internal controls. These samples came from a DNA bank of human DNA whose genotypes were previously confirmed by Taqman^TM^ and Sanger sequencing. Homozygous variant control samples were not available for *TPMT* rs1800462, *NUDT15* rs116855232, and the four *DPYD* SNPs since these genotypes are very rare in the population. Moreover, at the time of the method development, we did not have enough amount of DNA sample in our bank that matched with the genotype heterozygous for *DPYD* rs55886062 (low-frequency variant). Thus, this control was synthetized by performing a subcloning experiment as described in Supplementary Data 1.

The DNA samples used as internal control samples were chosen to get a balance between the lowest number of control samples to be analyzed and the need to be representative of each allelic combination of interest. Thus, we found a combination of only eight DNA samples suitable as internal controls of the 21 genotypes we had to discriminate in the patient samples. The genotypes of internal controls are shown in Supplementary Data 2, and their corresponding electropherograms are illustrated in Supplementary Data 3.

### 3.7. Validation of the Assay

Several parameters were assessed in order to validate the assay. Repeatability was assessed by genotyping internal control samples in duplicate six times on six independent experiments (6 different days). Accuracy was evaluated by the concordance of the genotype results of a cohort of human DNA analyzed with the new method in comparison with the results obtained from a previous analysis with the reference methods. Depending on the number of known genotype results available, the accuracy was assessed in at least 20 samples per gene of interest.

Robustness was checked by testing the influence of variation in the amount of DNA analyzed (from 1 to 500 ng) on the assay specificity and sensitivity. The influence of the quality of the DNA extracted was assessed by looking at the absorbance ratio (260/230 nm and 260/280 nm) of the DNA samples in our cohort. Intersample contamination was evaluated by inserting blank samples (water without DNA) between DNA samples. Besides, the stability of the mixed pool of diluted primers and fluorescent probes was checked for five freeze–thaw cycles (−20 °C ambient temperature). The genotyping results from six DNA samples were compared between the samples analyzed with a freshly prepared pool of primers and the same samples analyzed with the same pool frozen and thawed five times.

## 4. Discussion and Conclusions

Here, we propose a fast and cost-effective method to genotype in a multiplex assay of eight SNPs and one repetition of four genes involved in the pharmacological response of anticancer drugs. Instead of using different fluorescent primers to genotype each genetic polymorphism, we used universal primers as adapters so that we only had to order a single one couple of expensive fluorescent probes. Furthermore, the genotyping protocol can easily be extended with new SNPs by just ordering new standard primers. The method is therefore particularly suitable for low-throughput genotyping of well-known variants. This is a major advantage compared with similar multiplex assays such as SNaPshot^(R)^, which use fluorescent dNTP and more expensive reagents [8]. The performances of our assay were rigorously assessed in particular to confirm its accuracy and robustness. Accuracy was studied on 187 samples, all of which were correctly genotyped; therefore, the method is now routinely used in clinical practice in our center. However, more patients should be tested to get a complete measure of the sensitivity and specificity of the assay. We will collect further data on a prospective basis. A downside of the assay is that it is not designed to genotype tri-allelic variants since we use two fluorescent probes. Thus, we cannot exclude that some subjects are carriers of rare variants that would lead to false negative results. As in every PCR assay, we must also be aware that a polymorphism located in the 3′ region of primers could create a mismatch, leading to a failure of the amplification of the fragment of interest.

Besides, we showed that a minimal amount of DNA of 25 ng was needed in our assay to get interpretable results. This means that the sensitivity of this assay is lower than that of other existing multiplexing approaches (NGS, digital droplet PCR, etc.). However, in the case of the pharmacogenetic assay performed on constitutional DNA, we are not limited by the amount of DNA available in the samples since DNA is extracted from several millimeters of whole blood collected by venipuncture. Therefore, this assay is sensitive enough to the conditions of clinical use intended. A limitation of the protocol reported in the present study could be the number of variants that can be simultaneously studied. We multiplexed the analysis of 9 genetic variants. The instructions of the manufacturer of the multiplex PCR kit reagent used in our assay suggested that this approach is enough discriminant to genotype a panel of up to 16 variants. To be able to analyze a pharmacogenetic panel with a greater number of SNPs and samples, targeted sequencing would be more attractive. A comparison of usual pharmacogenetic methods regarding the criterion of cost and analytical performances is reported in Supplementary Data 4.

In conclusion, we developed a multiplex genotyping method that should make the analysis of pharmacogenes accessible to a large number of labs with a capillary sequencer by using inexpensive reagents and materials. Genotyping results can be returned in 1 day, which should be notably useful to optimize and individualize the treatment of many patients receiving anticancer drug therapy.

## Figures and Tables

**Table 1:** Genetic variants of interest included in the multiplex assay.

Gene	Name	Polymorphism (Location)	Frequency of the Minority Allele (%) ^a^	Consequence of the Allelic Variant on Protein Functionality
*TPMT*	Thiopurine S-methyltransferase	rs1800462 (exon 5)	1	Reduced
		rs1142345 (exon 10)	4	Reduced
		rs1800460 (exon 7)	3	Reduced
*NUDT15*	Nudix hydrolase 15	rs116855232	4	Reduced
*DPYD*	Dihydropyrimidine dehydrogenase	rs55886062 (exon 13)	0.1	Reduced (75%)
		rs3918290 (IVS 14)	0.5	No activity (Truncated) protein
		rs67376798 (exon 22)	0.7	Reduced (30%)
		rs75017182 (Hap B3–intron 10)	2.4	Reduced
*UGT1A1*	UDP glucuronosyltransferase family 1 member A1	rs8175347 (*28)	29–45	Reduced

^a^ According to [1] and [6].

**Table 2:** Accuracy of the assay.

Gene	Genetic Polymorphism	Genotype	Number of DNA Samples (Multiplex Method)	Concordance with Reference Method
*TPMT*	rs1800462(ex5)	WT	69	100%
		varHz	6	100%
	rs1800460(ex7)	WT	52	100%
		varHz	20	100%
		varHm	3	100%
	rs1142345(ex10)	WT	46	100%
		varHz	26	100%
		varHm	3	100%
*NUDT15*	rs116855232 C>T	WT	16	100%
		varHz	3	100%
*DPYD*	rs3918290(IVS14)	WT	133	100%
		varHz	5	100%
	rs55886062(ex13)	WT	128	100%
		varHz	1	100%
	rs67376798(ex22)	WT	125	100%
		varHz	4	100%
	rs75017182(int10)	WT	121	100%
		varHz	6	100%
*UGT1A1*	rs8175347 (*28)	WT	17	100%
		varHz	27	100%
		varHm	6	100%

WT: wild-type allele; varHz: heterozygous for the variant allele; varHm: homozygous for the variant allele.

**Table 3:** Influence of the amount of DNA analyzed on the performance of the analysis.

	DNA Amount							
Polymorphism	1 ng	5 ng	10 ng	25 ng	50 ng	100 ng	250 ng	500 ng
*TPMT* rs1800462	30	131	138	3064	4426	3455	>7500	6425
*TPMT* rs1142345	43	158	184	2999	4219	3301	>7500	7048
*TPMT* rs1800460	73	264	283	6385	7612	7105	3005	6198
*DPYD* rs3918290	N.D.	77	153	3151	4185	3198	>7500	7140
*DPYD* rs55886062	N.D.	129	171	2568	3633	2945	6599	7096
*DPYD* rs67376798	33	74	117	2467	3437	2578	6548	7232
*DPYD* rs75017182	36	141	163	3108	4625	3719	>7500	>7500
*NUDT15* rs116855232	73	313	371	7532	7596	7601	>7500	>7500
*UGT1A1* rs8175347	N.D.	61	81	1602	2335	1845	4339	5813

N.D.: undetermined. Results are expressed as peak intensity (height). A threshold of 100 intensity units is required to get a clear result. Intensity > 7500 is observed in the case of signal saturation.

**Table 4:** Stability of the pool of primers and probes ready for use.

Polymorphism	Mean Intensity with Fresh Mix	Mean Intensity with Mix FT5	Mean Bias (%) (FT5 vs. Fresh)
*TPMT* rs1800462	441	320	−31
*TPMT* rs1142345	2012	1229	−41
*TPMT* rs1800460	5453	3209	−44
*DPYD* rs3918290	2543	1352	−49
*DPYD* rs55886062	2081	1321	−38
*DPYD* rs67376798	1712	1097	−37
*DPYD* rs75017182	3086	1376	−56
*NUDT15* rs116855232	5583	2622	−54
*UGT1A1* rs8175347	2498	1165	−52
Mean	2823	1521	−45

(*n* = 6) Results are expressed as peak intensity (height). A threshold of 100 intensity units is required to get a clear result. Intensity > 7500 is observed in the case of signal saturation. FT5: experiment performed with the mixed pool of primers and probes used after five freeze–thaw cycles.

**Table 5:** Design of primers used in the assay.

Gene Position	Primer ID	Primer Sequences (5–3′)	Variants Detected	Amplicon Size (pb)	Primer Final Concentration in the Mix (pmol/µL)
TPMT rs1800462 (exon 5)	F-TPMT5-WT	**GTAAAACGACGGCCAGT**GTGTAAATGTATGATTTTATGCAGGCTTG	rs1800462	104	0.01
	F-TPMT5-var	**GTTTTCCCAGTCACGAC**GTGTAAATGTATGATTTTATGCAGGCTTC			0.01
	R-TPMT5	**GTTTCTT**GTATCCCAAGTTCACTGATTTCCAC			0.25
TPMT rs1800460 (exon 7)	F-TPMT7-WT	**GTAAAACGACGGCCA**GTCAAATTTGACATGATTTGGGATAGAAGAG	rs1800460	173	0.01
	F-TPMT7-var	**GTTTTCCCAGTCACGAC**CAAATTTGACATGATTTGGGATAGAAGAA			0.01
	R-TPMT7	**GTTTCTT**AGTCTAAGCTGATTTTCTAGAACCC			0.7
TPMT rs1142345 (exon 10)	F-TPMT10-WT	**GTAAAACGACGGCCAGT**GGAATTGACTGTCTTTTTGAAAAGTGATA	rs1142345	152	0.01
	F-TPMT10-var	**GTTTTCCCAGTCACGAC**GGAATTGACTGTCTTTTTGAAAAGTGATG			0.01
	R-TPMT10	**GTTTCTT**CCATTACATTTTCAGGCTTTAGCA			1
DPYD rs3918290 (IVS 14)	F-DPYD-IVS14-WT	**GTAAAACGACGGCCAGT**CTCTTGTTTTAGATGTTAAATCACACCTAC	rs3918290	204	0.01
	F-DPYD-IVS14-var	**GTTTTCCCAGTCACGAC**CTCTTGTTTTAGATGTTAAATCACACCTAT			0.01
	R-DPYD-IVS14	**GTTTCTT**GTTTCCCCCAGAATCATCCG			0.7
DPYD rs55886062 (exon 13)	F-DPYD-ex13-WT	**GTAAAACGACGGCCAGT**TCCAGCTTCAAAAGCTCTTAGAA	rs55886062	226	0.01
	F-DPYD-ex13-var	**GTTTTCCCAGTCACGAC**TCCAGCTTCAAAAGCTCTTAGAC			0.01
	R-DPYD-ex13	**GTTTCTT**CCAAGTATTGGTTTGTATTTTGCAG			1
DPYD rs67376798 (exon 22)	F-DPYD-ex22-WT	**GTAAAACGACGGCCAG**TCCACAGTTGATACACATTTCTCCAT	rs67376798	254	0.01
	F-DPYD-ex22-var	**GTTTTCCCAGTCACGAC**CCACAGTTGATACACATTTCTCCAA			0.01
	R-DPYD-ex22	**GTTTCTT**CCAGTCTCCCAAGTTAATATAATGC			1
DPYD rs75017182 (Hap B3-intron 10)	F-DPYD-int10-WT	**GTAAAACGACGGCCAGT**CTGAATATGGAGGTGAAAATCACAGC	rs75017182	274	0.01
	F-DPYD-int10-var	**GTTTTCCCAGTCACGAC**CTGAATATGGAGGTGAAAATCACAGG			0.01
	R-DPYD-int10	**GTTTCTT**GGATATGAATGCTTCTCCTCATGG			0.7
UGT1A1 rs8175347 (*28)	F-UGT1A1-WT	**GTAAAACGACGGCCAGT**TGTATCGATTGGTTTTTGCCATATATATATACATA	rs8175347	343–345	0.01
	F-UGT1A1-var	**GTTTTCCCAGTCACGAC**TGTATCGATTGGTTTTTGCCATATATATATACATATA			0.01
	R-UGT1A1	**GTTTCTT**GGCACAGGGTACGTCTTCAA			1
NUDT15 rs116855232	F-NUDT15-WT	**GTAAAACGACGGCCAGT**CCAGCTTTTCTGGGGATTGC	rs116855232	293	0.01
	F-NUDT15-var	**GTTTTCCCAGTCACGAC**CCAGCTTTTCTGGGGATTGT			0.01
	R-NUDT15	**GTTTCTT**TCTCAAGTACTGGCTGAAAGAGT			0.7

F: forward; R: reverse; WT: wild type; var: variant. **GTAAAACGACGGCCAGT:** universal M13 sequence (−20) added to the 5′ flanking forward wild-type primers. **GTTTTCCCAGTCACGAC:** M13 sequence (−40) added to the 5′ flanking forward variant primers. **GTTTCTT:** “pigtail” added to the 5′ flanking reverse primers.

## Supplementary Materials

Supplementary materials can be found at https://www.mdpi.com/1422-0067/21/24/9650/s1. Supplementary Data 1: Synthesis of control DNA for the variant DPYD rs55886062, Supplementary Data 2. Genotype of internal quality control DNA samples, Supplementary Data 3: Representative electropherogram of QC samples, Supplementary Data 4: Comparison of PharmFrag assay with usual pharmacogenetic methods according to cost, time of analysis, and analytical parameters and performances of the assays.
