# Supplementary material for: PharmFrag: An Easy and Fast Multiplex Pharmacogenetics Assay to Simultaneously Analyze 9 Genetic Polymorphisms Involved in Response Variability of Anticancer Drugs
Source: Int J Mol Sci. 2020 Dec 17;21(24):9650. doi: 10.3390/ijms21249650 (PMC7766892; doi:10.3390/ijms21249650)
Supplement: Supplementary file 1 [file ijms-21-09650-s001.pdf]

International Journal of *Molecular Sciences*

Supplementary Data 1: Synthesis of control DNA for the variant *DPYD* rs55886062.

First, the genomic region containing the exon of interest was amplified by PCR (CloneAmp HiFi PCR from Takara Bio, Saint-Germain-en-Laye, France) using wild-type and variant DNA and the primers (primer pairs: primer sequences (5'–3') pF-IF_DPYDex13 TAAGAAGTGCAGGATCCCTTACCTTATCAAGAGAGAAAGTTTTGGTG, pR-IF_DPYDex13 GTCAAAACAAGACGCGTCAGACTGTTGTAATCTATATTAATTCGGATGCT). The 5' flanking primer region was designed in complementarity with the cloning site's pCAs2 vector. The PCR products were inserted into a previously linearized pCAS2 (BamH1/Mlu1 digestion) by an In-Fusion® HD Cloning kit (Takara Bio). NEB® 5-alpha Competent *Escherichia coli* was used to amplify the two constructs. Finally, the inserts were sequenced to ensure that no unwanted mutation was introduced during PCR or cloning. An equimolar pool of the two plasmids carrying the wild-type or a mutant genomic insert for rs55886062 was made to obtain a heterozygous mutated control.

Supplementary Data 2. Genotype of internal quality control DNA samples.

QC Name	TPMT exon 5	TPMT exon 10	TPMT exon 7	DPYD IVS 14	DPYD exon 13	DPYD exon 22	DPYD intron 10	NUDT15	UGT1A1
**QC1**	WT	WT	WT	WT	WT	WT	WT	WT	WT
**QC2**	varHz	WT	WT	WT	WT	WT	WT	WT	WT
**QC3**	WT	varHz	varHz	WT	WT	WT	varHz	WT	varHz
**QC4**	WT	varHm	varHm	WT	WT	WT	WT	WT	WT
**QC5**	WT	WT	WT	varHz	WT	WT	WT	WT	WT
**QC6**	-	-	-	-	varHz	-	-	-	-
**QC7**	WT	WT	WT	WT	WT	varHz	WT	WT	varHm
**QC8**	WT	WT	WT	WT	WT	WT	WT	varHz	varHz

QC: quality control; WT: wild-type allele; varHz: heterozygous for the variant allele; varHm: homozygous for the variant allele.

Supplementary Data 3: Representative electropherogram of QC samples.

QC1

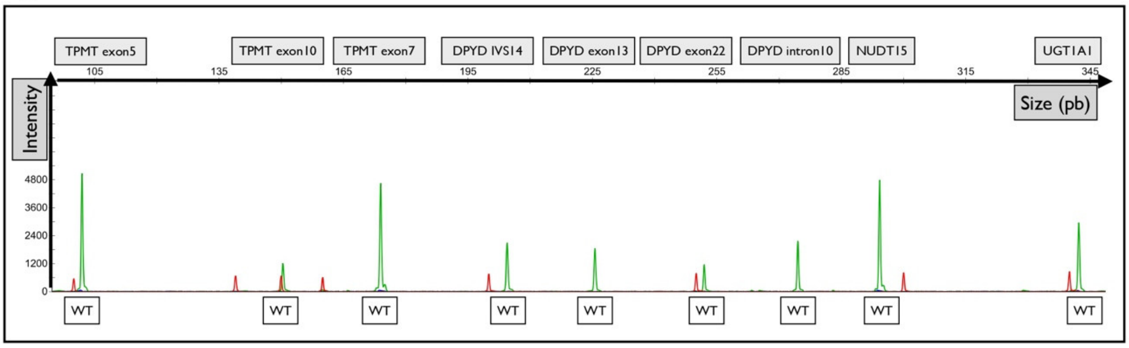

QC2

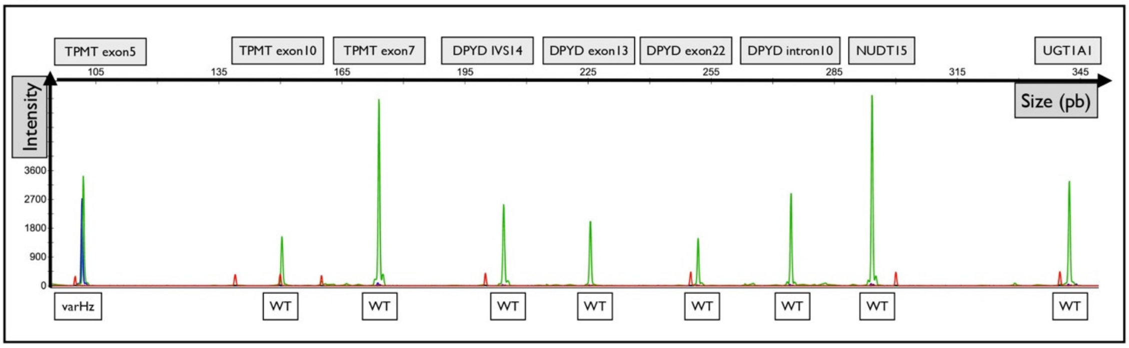

QC3

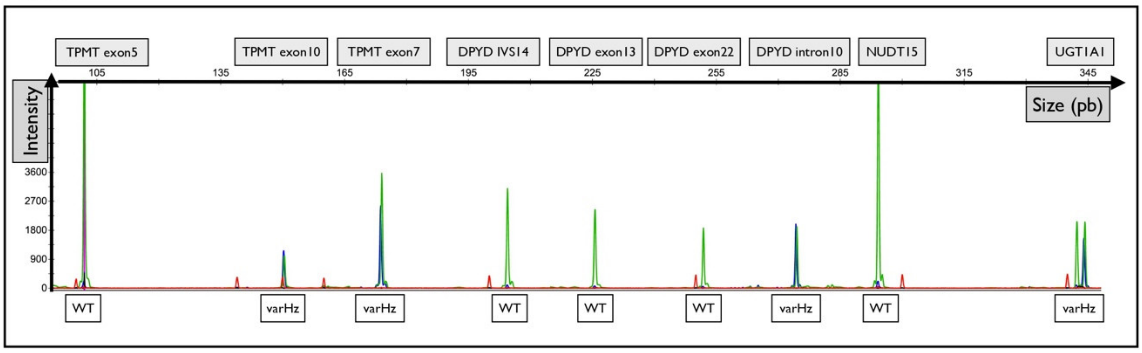

QC4

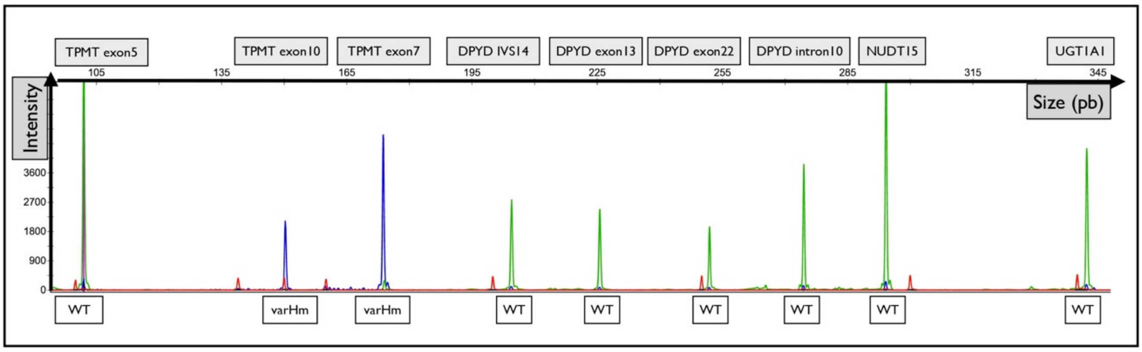

QC5

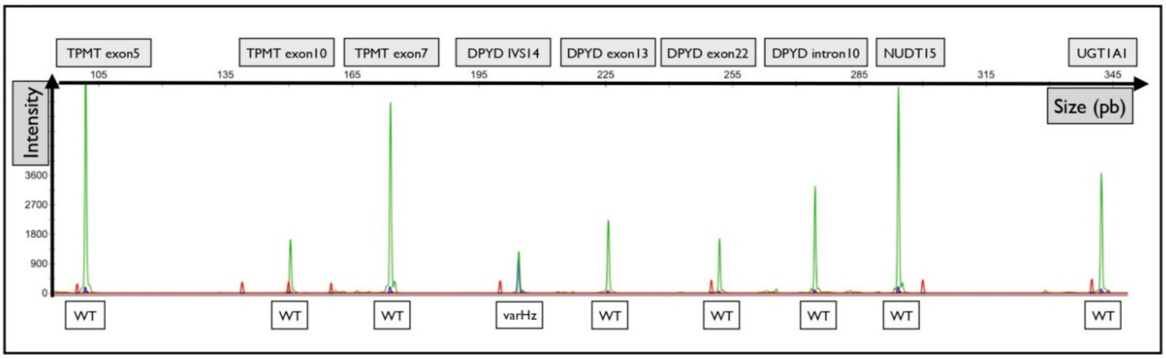

QC6

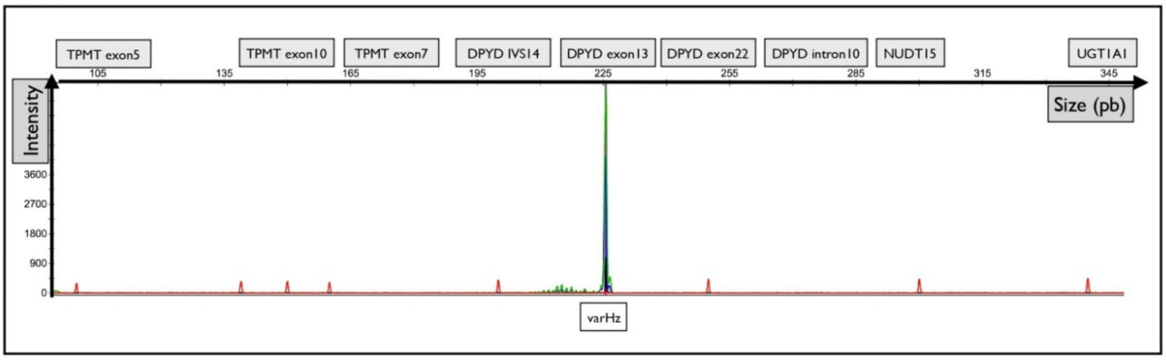

QC7

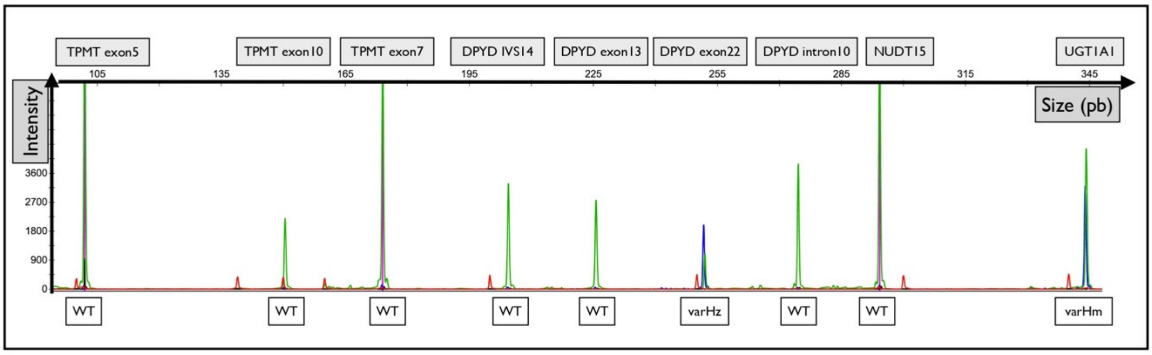

QC8

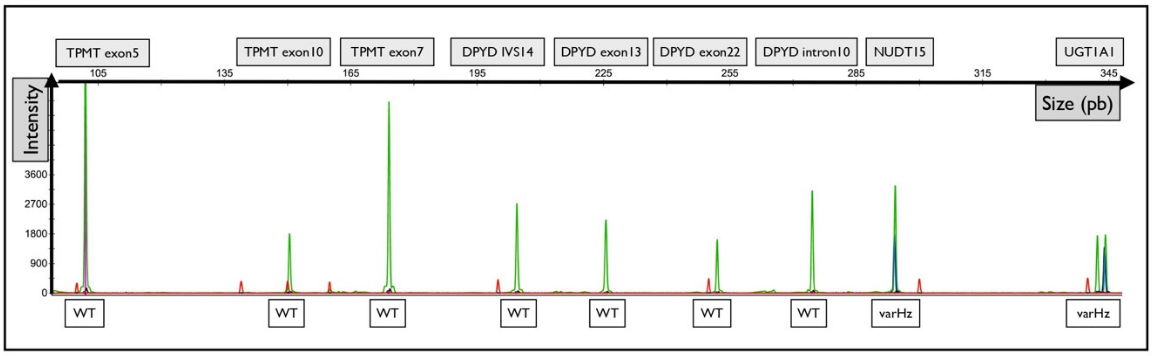

Supplementary Data 4: Comparison of PharmFrag assay with usual pharmacogenetic methods according to cost, time of analysis, and analytical parameters and performances of the assays.

**Parameters**		**PharmFrag**	**TaqMan® Genotyping Assays**	**Sanger Sequencing**	**Next-Generation Sequencing**
Cost(1)	Price/10 samples for 9 variants (euros)	55	320	1440	2500
	Price per hotspot (euros)	6	36	16	5
Time	Time of technical workload/10 samples (hours)	1	2	4	4
	Time in automated device/10 samples (hours)	3	2	12	24
	Time of analysis workload/10 samples (hours)	0.5	1	2	1
	Total (hours)	4.5	5	18	29
Analytical parameters	DNA sample amounts needed (ng)	50	10	50	5
	Stability of primer and fluorescent probes	Good	Good	Excellent(2)	Good
	Specificity	Good	Good	Excellent	Excellent
	Sensitivity	Good	Good	Good	Excellent
	Throughput	Medium	Medium	Low	High

(1) These data are estimations based on our center experiences and usual literature reports. Prices are evaluated based on cost of reagent and consumables. They do not include personal cost or cost of equipment acquisition since this part depends on each laboratory policy. (2) No fluorescent dye needed. For a moderate number of samples to be analyzed (moderate throughput), PharmFrag is a costless and the most cost-effective method. PharmFrag and TaqMan® assays are the fastest since results can be available within a working day.

© 2020 by the author. Licensee MDPI, Basel, Switzerland. This article is an open access article distributed under the terms and conditions of the Creative Commons Attribution (CC BY) license (http://creativecommons.org/licenses/by/4.0/).
